# Increased fluvial runoff terminated inorganic aragonite precipitation on the Northwest Shelf of Australia during the early Holocene

**DOI:** 10.1038/s41598-019-54981-7

**Published:** 2019-12-04

**Authors:** Maximilian Hallenberger, Lars Reuning, Stephen J. Gallagher, Stefan Back, Takeshige Ishiwa, Beth A. Christensen, Kara Bogus

**Affiliations:** 1 Energy and Mineral Resources Group (EMR), Geological Institute, RWTH Aachen University, Aachen, Germany; 2 Institute of Geosciences, CAU Kiel, Kiel, Germany; 3 School of Earth Sciences, University of Melbourne, Melbourne, Australia; 4 National Institute of Polar Research, Tokyo, Japan; 5 School of Earth and Environment, Rowan University, Glassboro, New Jersey United States; 6 Camborne School of Mines, University of Exeter, Exeter, United Kingdom

**Keywords:** Palaeoceanography, Palaeoclimate, Sedimentology

## Abstract

Inorganic precipitation of aragonite is a common process within tropical carbonate environments. Across the Northwest Shelf of Australia (NWS) such precipitates were abundant in the late Pleistocene, whereas present-day sedimentation is dominated by calcitic bioclasts. This study presents sedimentological and geochemical analyses of core data retrieved from the upper 13 meters of IODP Site U1461 that provide a high-resolution sedimentary record of the last ~15 thousand years. Sediments that formed from 15 to 10.1 ka BP are aragonitic and characterised by small needles (<5 µm) and ooids. XRF elemental proxy data indicate that these sediments developed under arid conditions in which high marine alkalinity favoured carbonate precipitation. A pronounced change of XRF-proxy values around 10.1 ka BP indicates a transition to a more humid climate and elevated fluvial runoff. This climatic change coincides with a shelf-wide cessation of inorganic aragonite production and a switch to carbonate sedimentation dominated by skeletal calcite. High ocean water alkalinity due to an arid climate and low fluvial runoff therefore seems to be a prerequisite for the formation of shallow water aragonite-rich sediments on the NWS. These conditions are not necessarily synchronous to interglacial periods, but are linked to the regional hydrological cycle.

## Introduction

**Figure 1:**
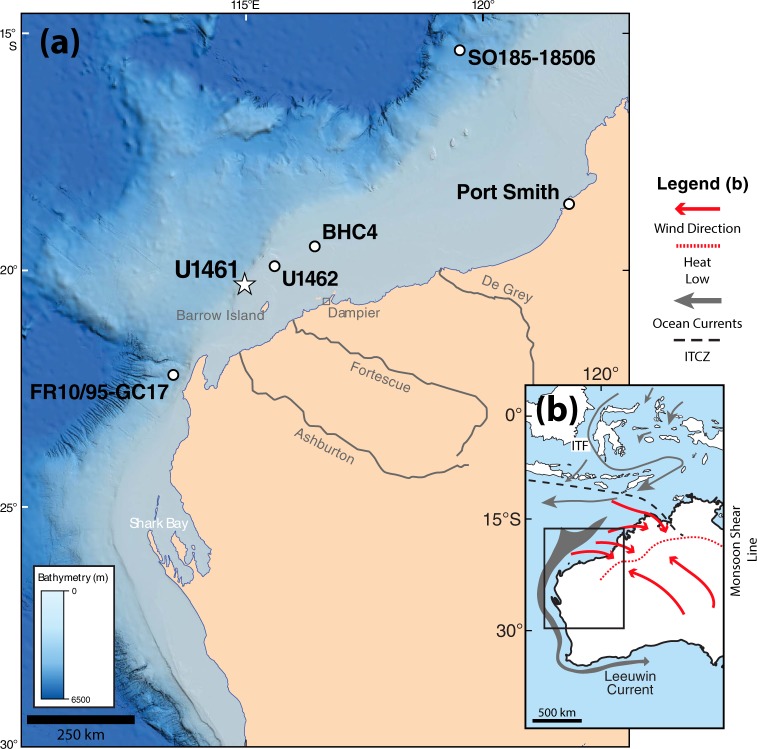
(**a**) Location map of the NWS showing Site U1461 and other locations referred to in text (white dots). The figure is modified after Figure F1 in Gallagher *et al*.^40^ (http://publications.iodp.org/proceedings/356/356title.html). The original figure is licensed under CC BY 4.0 (https://creativecommons.org/licenses/by/4.0/). (**b**) Present-day oceanography of Australia/Indonesia^56^. Displayed in red are the average wind direction of monsoonal and trade winds during January as well as the monsoon shear line in northern Australia^57^. The average position of the Intertropical Convergence Zone (ITCZ) is displayed during austral summer (January)^58^. The base map was redrawn using the vector graphics editor Adobe Illustrator (Map data ©2018 Google).

The modern Northwest Shelf of Australia (NWS) is a distally steepened carbonate ramp which stretches between ~13° and 21° S (Fig. 1)^1^. It forms an extensive carbonate factory which rivals the Bahamas or the Persian Gulf in size and represents an important, yet poorly understood, analogue to ancient carbonate ramps.

Holocene sedimentation across the NWS is limited and primarily occurs at the inner ramp below 50 metres of water depth (mwd) and along a small pelagic ridge at approx. 140 mwd^1^. The Holocene sediments are largely calcitic and mainly consist of benthic and planktic skeletal fragments^1,2^. Coral fragments contribute locally to the sediment around fringing and patch reefs on the inner ramp, e.g. southwest of Dampier^3^, or around isolated shelf edge reefs^4^.

Across the mid to outer ramp sediments are dominated by ooids and aragonite needle mud^1,2,5^. Both were dated to have formed shortly after the Last Glacial Maximum (LGM) and are currently stranded at the sea-floor in water depths from 50 to 100 meters and 120 to 200 meters respectively^1,5^. A rare exception of Holocene ooid formation can be found near Port Smith (Fig. 1), where suitable conditions led to an 800 year-long pulse of ooid formation along a tidal inlet^6^.

The distribution of present-day seafloor sediment therefore implies that the composition, fabric, and mineralogy of the NWS carbonates significantly changed during the last glacial-interglacial transition. However, until recently, subsurface data of the Pleistocene-Holocene transition on the NWS was limited. Core samples from industry wells do not resolve this stratigraphic interval sufficiently^7^, whereas sediment cores off the NW Australian margin do not record sedimentary processes on the shelf (Fig. 1)^8–11^.

In 2015, this gap in data availability was addressed by IODP Expedition 356, which cored six sites along the NWS. This study focuses on the upper 13 meters of IODP Site U1461 applying a combination of petrographical methods and X-ray fluorescence (XRF) analyses derived proxy records. Variations in terrestrial input are based on titanium (Ti) which originates from lithogenic sources. Titanium is normalized against calcium (Ca) which is controlled by the production of marine carbonates^10,12,13^. We further used the ratio of zirconium (Zr) to aluminium (Al) and potassium (K). Zirconium is transported by aeolian processes^11,14^ while aluminium and potassium are primarily transported by fluvial processes^10,11^. The ratio between those elements therefore represents a proxy of fluvial runoff and dust flux.

Analyses of the integrated geochemical and sedimentological dataset allows us to better understand depositional and mineralogical changes in the transition from the late Pleistocene to the Holocene.

## Climate and Oceanography

The climate along the NWS is mostly arid with seasonal rainfall during the Australian Summer Monsoon^15^. This rainfall sources several river systems, most prominently the De Grey and the Fortescue river, which transport siliciclastic material to the shelf (Fig. 1). The variability in terrigenous runoff has been previously used as a proxy for monsoonal strength over time, revealing that the Australian Summer Monsoon was weakened during glacial periods^10,16,17^. The oceanography is characterized by south-flowing shallow currents, which transport warm and low-salinity water along the Indonesian Throughflow to the NWS (Fig. 1). These currents, which feed around cape range into the Leeuwin Current, are thought to be severely weakened during glacials^7,18–20^.

## Results

**Figure 2:**
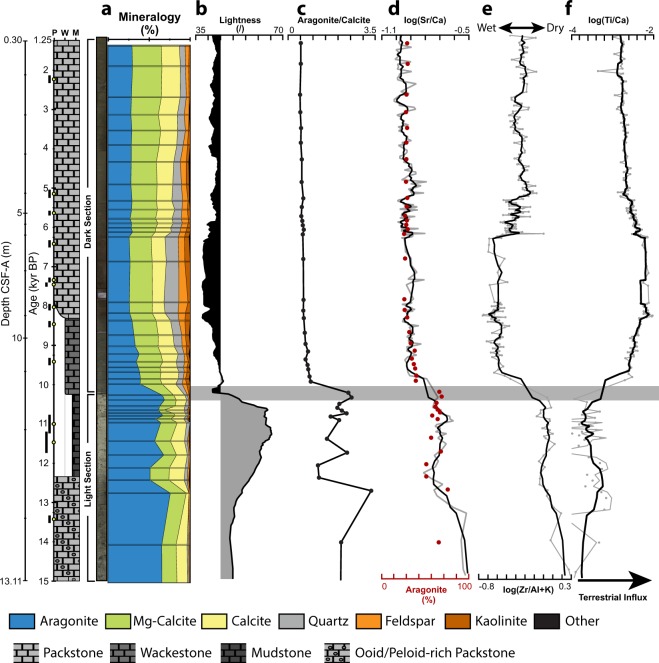
Chronostratigraphic overview of collected data from the upper 13 meters (15 kyr) of Hole U1461C including core images of the “light” and “dark” section. Age measurements and 95% density region are indicated by yellow dots and a black bar along the linear time axis. Core images have been stretched and compressed according to the age-model. The grey bar indicates the timing of the upward transition from inorganic aragonite-rich sediments to biogenic calcite-rich sediments. (**a**) XRD-derived cumulative bulk mineralogy; (**b**) Reflectance (lightness) values of hole C. The cut-off value chosen to divide the “light” and “dark” section is equal to 45 and represents the point of highest inflection within the dataset; (**c**) Aragonite to calcite ratio; (**d**) Aragonite content and calibrated XRF-derived log ratio of (Sr/Ca); (**e,f**) XRF-derived log ratios of (Zr/Al + K) and (Ti/Ca).

The strata of Site U1461 can be preliminarily subdivided by colour into an upper “darker” and a lower “lighter” unit (Fig. 2). This separation is supported by the lightness log, which generally shows low values in the “dark” section (<45) and high values in the “light” section (>45) (Fig. 2). The lighter part of the succession, which formed from 15 to 10.1 ka BP (13–11.5 m) is unlithified, with a light grey to grey colour. Carbonate mineralogy within this unit is dominated by aragonite (51–76%, mean = 64%) with lesser high-Mg calcite (6–26%, mean = 18%) and low-Mg calcite (1–25%, mean = 13%). Generally high aragonite to calcite ratios (>2) typify “light” sediments (Fig. 2). Terrestrial influx during this time was relatively low, as can be seen by the negligible amounts of siliciclastics present (0–7%, mean = 4%) and the low log ratios of (Ti/Ca) (Fig. 2). Log ratios of (Zr/Al + K) are elevated in this unit, indicating a dry climate where terrestrial material is predominantly derived from aeolian sources. A slight shift towards increased terrestrial influx can be found within the “light” section at around 12.5 ka BP (~12.7 m). This shift is accompanied by a minor decrease in aragonite content as resolved by changes in log(Sr/Ca) (Fig. 2).

**Figure 3:**
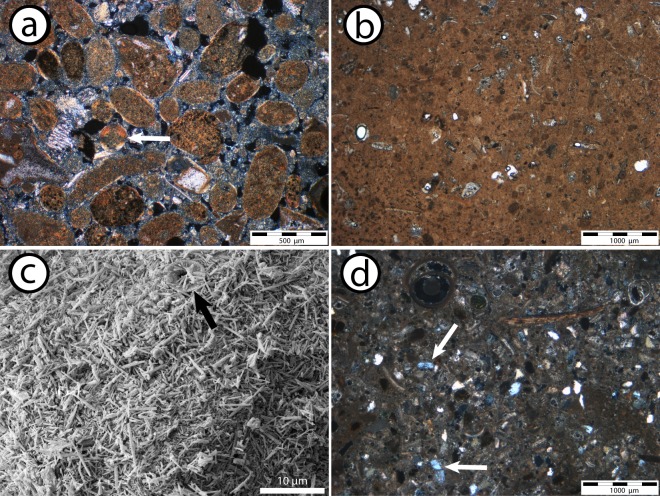
Representative thin-section and SEM images of sediments found within the upper 13 meters of Site U1461. **(a)** Sediments formed from 15 ka BP to ~12 ka BP contain abundant non-skeletal grains, including peloids and superficial ooids (white arrow), U1461C-2H-3W-93 cm, 12.34 m CSF-A, XPL. **(b)** Sediments formed between ~12 ka BP to 10.1 ka BP are composed of an aragonitic micrite and minor amounts of skeletal fragments, U1461C-2H-3W-43 cm, 11.84 m CSF-A, PPL **(c)** Micrite found within aragonite-rich sediments consists almost entirely of small (2–4 µm) needles. Rare coccolith plates can also be found (black arrow), U1461C-2H-3W-43 cm, 11.84 m CSF-A. **(d)** “Dark” sediments which formed between 10.1 ka BP till present are rich in skeletal grains as well as detrital grains, including quartz (white arrows), lithic fragments, and feldspar, U1461C-2H-2W-43 cm, 10.34 m CSF-A, XPL.

Based on their sedimentary composition the “light” unit can be further subdivided into a non-skeletal grain-rich wacke- to packstone which transitions at around 12 ka BP (~12.3 m) into a wacke- to mudstone up section (Fig. 2). The non-skeletal grain-rich wacke- to packstone contains large amounts of superficial ooids and peloids (Fig. 3). These grains comprise up to 50% of the bulk sediment and are predominantly composed of aragonite (>85%). Bioclasts are rare and include benthic foraminifers (10%), planktic foraminifers (<5%), bivalve fragments (<5%), and echinoderms (<5%). At around 12 ka BP non-skeletal grain-rich facies transitions into a wacke- to mudstone (Fig. 2). These sediments predominantly (~80%) consist of small (2–4 µm) aragonite needles (Fig. 3). Ooids are absent and peloids are rare (<5%). Bioclasts, which on average represent 20% of the sediment, are more abundant and diverse than within the underlying non-skeletal grain-rich wacke- to packstone. Bioclasts include planktic (5%) and benthic (5%) foraminifera. Minor amounts of echinoderms, bivalve fragments, ostracods, gastropods, and bryozoans are each present in trace amounts (<5%). Additionally, rare (<5%) coccolith plates occur in the mud fraction (<64 µm) (Fig. 3).

At 10.1 ka BP (11.5 m) the upper, darker part of the core starts and the aragonite-rich facies transition into an unlithified olive-grey to dark-grey wacke- to packstone (Fig. 2). This transition is accompanied by major changes in mineralogy, XRF-ratios and sediment composition. High-Mg calcite (24–36%, mean = 30%) and low-Mg calcite (14–23%, mean = 20%) become more abundant while aragonite content decreases (26–40%, mean = 31%) (Fig. 2). The relative abundance of siliciclastics (12–32%, mean = 19%) increases markedly, indicating an overall increase of riverine runoff associated with an increase in log ratios of (Ti/Ca) and decreasing log ratios of (Zr/Al + K). This transition is followed by a tableau in runoff ranging from 10 to 6.3 ka BP. At around 6.3 ka BP XRF-ratios and siliciclastic content suggest a return to more arid conditions with decreasing terrestrial input (Fig. 2). This change is expressed as a small shift towards higher log(Zr/Al + K) and lower log(Ti/Ca) values from 6.3 to 6 ka BP. Afterwards values remain mostly stable with a slight but gradual trend towards a drier climate and reduced runoff. These trends are not reflected in the carbonate mineralogy variability, with constant low (~0.5) aragonite to calcite ratios above the lighter to darker unit transition at 10.1 ka BP (Fig. 2).

The mineralogical changes at ~10.1 ka BP coincide with a considerable change in sediment composition. Unlike prior to 10.1 ka BP, ooids, peloids, and aragonite needles are entirely absent. Sediments are instead composed of skeletal fragments, which consist of planktic (10%) and benthic (10%) foraminifers, and gastropod shells (20%). Echinoderms, sponge needles, ostracods, bryozoans, and bivalve fragments all occur in trace amounts (<5%) (Fig. 3). The majority of aragonite found within the darker section is bound to pteropod and heteropod (gastropod) shells. Other gastropods are present as well, however they are relatively rare. Aragonite is further present within the mud fraction (<64 µm), which was measured separately on two samples, revealing a composition which is equivalent to the bulk mineralogy. It is important to note that the mud fraction within this section does not contain any needles.

## Discussion

The occurrence of ooids on the NWS as far back as Marine Isotope Stage 18 (MIS 18) was linked to phases of aridity^7,21^. However, these studies were lacking the stratigraphic control to unravel the relationship between climate, sea-level change and inorganic carbonate precipitation. The upper 13 meters of IODP Site U1461 show evidence for a rapid change in primary carbonate composition, from a system which is dominated by non-skeletal grains and aragonite needle mud, to a biogenic calcite-rich system. This shift happened around 10.1 ka BP with geochemical proxies indicating that the observed change was associated with increased riverine runoff (Fig. 2). The core-based interpretations therefore corroborate previous observations that postulated dry glacials and humid interglacials along NW-Australia^7,9–11^ (Supplementary Fig. S1). Following the last glacial (MIS 2), humid conditions became established by a southward migration of the Intertropical Convergence Zone (ITCZ) and the post-glacial onset (intensification) of the Australian Summer Monsoon^9–11^. The progressive southward movement of the ITCZ led to a distinct NW-SE trend throughout the region, with humid conditions occurring between ~14–15 ka BP in the Banda Sea^22^, at 13 ka BP in the Timor Strait, and at 11.6 ka BP at the north-eastern parts of the NWS^11^ (SO185–18506, Fig. 1). At Site U1461 this trend was verified with a proposed onset of humid conditions at around 11.5 ka BP^17^ (Supplementary Fig. S1). The new, better resolved age model for this site suggests a later onset of humid conditions at ~10.1 ka BP (Supplementary Table S1, Fig. 2).

The origin of the aragonite-rich sediments formed between 15 to 10.1 ka BP is interpreted to be primarily inorganic. Ooids are widely recognized to form by the precipitation of carbonate around a pre-existing nucleus. This process preferentially takes place in agitated shallow and warm water, when alkalinity is sufficiently high for abiotic carbonate precipitation to occur^1,21,23^. Peloids, which are the other main component of non-skeletal grain-rich sediments, need to undergo an early “*in-situ*” cementation to be preserved within the sedimentary record. Similar to the formation of ooids, this cementation demands favourable physicochemical oceanographic conditions^24,25^. These conditions are predominantly found in tropical, shallow-water marine environments, where elevated temperatures and alkalinities enable an early “hardening” of peloids^24^. Notably, this process may also take place in comparatively deep water, as has been recently shown for peloids found offshore SW-Australia^26^. However, the fact that peloids often form the nucleus for ooids at Site U1461 strongly indicate that they formed within a shallow, tropical environment (Fig. 3).

Aragonite needle mud found stranded at the present day NWS seafloor is interpreted to have formed due to the inorganic precipitation via “whiting” events, similar to processes observed at the present day Bahamas^5^. This interpretation is based on similarities in appearance and isotope signature between aragonite needles found at the NWS and those presently forming in the Bahamas^5,27^. While the exact origin of “whitings” is still debated, most theories agree on the necessity of elevated aragonite saturation states and high ocean water residence times for such processes to take place^27–29^.

XRF proxy data indicates that prior to 10.1 ka BP precipitation of inorganic aragonite was facilitated by a dry climate, where a lack of fluvial runoff led to a high carbonate saturation and ocean water alkalinity across the shelf (Fig. 2). Following ~ 6 ka BP XRF proxy data suggests a return to more arid conditions (Fig. 2) This trend is well documented for NW-Australia, with deep-water records matching records from the NWS (Supplementary Fig. S1)^11,17^. At Site U1461, the return to more arid conditions did not result in a re-emergence of inorganic aragonite production. We therefore conclude that fluctuations in aridity are not necessarily expressed in incremental changes of inorganic aragonite production. Instead, a sufficiently low fluvial runoff facilitated by high aridity appears to be a requisite for inorganic aragonite precipitation to take place at the NWS. Ooids/peloids and aragonite needle mud are interpreted to have formed simultaneously, albeit within different water depths. Ooids develop within very shallow (<5 mwd) and agitated water^23^. They therefore represent the most proximal sedimentary components found at Site U1461. However, the co-occurrence of open marine planktic foraminifers indicates that ooids at Site U1461 were subsequently transported to deeper water. A similar redeposition towards deeper waters has been reported for ooids formed during the last glacial at Maui Nui (Hawaiian Islands)^30^.This is in agreement with the proposed age of the ooid-rich section of 15 to 12 ka BP, a time during which Site U1461 resided within estimated water depths of 30 to 70 meters^31,32^.

Present-day whiting events typically occur within shallow water depths^33,34^. It was suggested that the needles were transported offshore and eventually settled into deeper water below wave base^5^. This is indicated by the present-day distribution of stranded aragonite-rich sediments, where ooid/peloids reside within shallower water depths than aragonite needle mud^1,5^. At Site U1461 the fossil assemblage of the aragonite needle mud facies is more pelagic-rich compared to ooid/peloid wacke- to packstone, further indicating a higher paleo-water depth (Fig. 3). This interpretation fits the ongoing post-glacial sea-level rise, which puts the aragonite mud-rich section into deeper water than the underlying older ooid/peloid-rich section^32,35^.

After 10.1 ka BP deposition of inorganic aragonite ceased at Site U1461. This cessation is likely representative for the NWS as a whole, since there is no modern production of aragonite-rich sediments in shallow water depths^1–3,5^. However, non-skeletal grains and aragonite mud similar to those described for Site U1461 are present at the seafloor of the mid to outer ramp^1,5^. These sediments are stranded and have been dated to have formed between 19.8 to 19 and 15.4 to 12.7 ka BP for aragonite needle mud and ooids respectively^1,5^. Our data indicates that the production of aragonite-rich sediments persisted until 10.1 ka BP, and therefore significantly longer than previously thought. This has direct implications for previous interpretations, which attributed an arrest of ooid production to the intensification of the Leeuwin Current^1^. This interpretation is based on the proposed coeval onset (intensification) of the Leeuwin Current at around 12 ka BP and the age of the youngest stranded ooids dated for the present day seafloor^1,9,20^. However, estimates of the timing of Leeuwin Current intensification predate the arrest of inorganic aragonite production at Site U1461 by around two thousand years^19,20,36^. Meanwhile, the XRF-climate proxies show that fluvial influx and carbonate mineralogy changed synchronously over the course of about 500 years implying a strong control of the hydrological cycle on aragonite supersaturation on the shelf (Fig. 2). This change from aragonite to calcite dominated sedimentation might have been facilitated by a general decrease in aragonite supersaturation of surface waters in the tropics during this time due to the deglacial rise in atmospheric CO_2_^37^.

However, despite of this general trend, aragonite is the dominant tropical carbonate phase formed during the Holocene in many parts of the world, most notably the Bahamas^38,39^. This implies the importance of regional climatic factors for the formation or cessation of aragonite precipitation at the NWS. The projected anthropogenic increase in CO_2_ and the associated global decline in aragonite supersaturation might increase the sensitivity of carbonate systems worldwide to regional climate forcing. Similar shelf wide reduction of inorganic or bio-induced aragonite precipitation in favour of biogenic calcite production might therefore become more common in the future.

## Conclusion

The observed change in carbonate composition and mineralogy is closely connected to the prevailing climate conditions. During an arid phase until 10.1 ka BP, inorganic aragonite was formed and deposited on the shelf. These sediments are dominantly composed of aragonite needle mud, ooids and peloids. After 10.1 ka BP humidity and fluvial runoff increased rapidly and inorganic precipitation of aragonite ceased completely. The timing of this change does not show a clear relationship with important steps in the deglacial flooding history of the shelf. Instead the increased fluvial runoff associated with a gradual southward shift of the ITCZ and the onset of the Australian Summer Monsoon is thought to be the primary control for the termination of inorganic aragonite production. Following 10.1 ka BP sedimentation across the NWS is dominated by calcitic skeletal fragments. Residual aragonite is bound to biogenic sources, most notably gastropod shells. The presented dataset therefore demonstrates that short-term changes in regional climate and associated shallow-water ocean alkalinity can act as a strong control on carbonate deposition across the entire shelf system. Future ocean acidification likely will make more shelf regions in the tropics susceptible for the influence of similar regional climatic processes.

## Methods

### Material and samples

Site U1461 (20° 12.863′S, 115° 3.950′E) is situated in a water depth of 127 m about 100 km northwest of Barrow Island in the Northern Carnarvon Basin on the shelf edge of an outer ramp (Fig. 1)^1,40^. A total of four holes have been drilled in close vicinity, three of which (U1461A–U1461C) cored the studied depth segment from 0 to 13 m depth. Core recovery within this section is above 95% in general and 100% considering overlap between single holes. Drilling disturbances are rare, and bioturbation is described as being low to absent^41^. Samples have been primarily selected from Hole C and supplemented from Hole A and B were drilling disturbance was high or core material was missing. The depth scale is therefore adopted from hole C and expressed as CSF-A as defined by the IODP Depth Scales Terminology^42^. The depth of samples derived from Hole A and B are matched to Hole C by the common meters composite depth (CCSF)^43^. Sample denotation follows the IODP guideline outlined as Site/Hole-Core-Section-distance from top^43^.

### Sedimentary composition and mineralogy

Sediment composition and texture has been determined by thin-section analysis (n = 8), using a petrographic microscope (Olympus BH-2) and a Scanning Electron Microscope (Supra55, Zeiss). The relative abundance of different microfossils was estimated using a visual percentage chart^44^. The resulting values are integrated into an existing dataset including 23 smear slides (shipboard data, IODP Exp. 356). Optic data was complemented by the shipboard derived lightness log, which is a unitless spectrophotometric parameter derived from the reflectance of visible light on split cores^43^. Sample mineralogy was analysed using a Siemens D5000 x-ray diffractometer. A total of 43 samples were oven-dried, grounded and mounted on sample holders. Two additional samples have been sieved for their mud (<63 µm) fraction which was then measured separately. The measurements were conducted over an angle field of 66° (4–70°) with a step size of 4 * 10^-30^ per second. Identification and quantification of different mineral phases was achieved by standard Rietveld refinement using the software DIFFRAC EVA (ver. 8.0, Bruker) and Profex (ver. 3.14.0). Non-destructive semi-quantitative determination of single component mineralogy was achieved by 2-D XRD measurements^45^ (D8-Bruker, resident time per spot = 10 Minutes). Measured components include ooids, peloids and a variety of different bryozoa.

### XRF analyses

X-ray fluorescence scanning was performed with an Avaatech XRF core scanner at the JRSO XRF Core Scanning Facility, located at the Gulf Coast Repository in the Texas A&M University Research Park. Each core section was covered with a 4 μm-thick ultralene film to avoid contamination and desiccation. XRF scanner measurements were carried out with a generator setting of 9 kV and 30 kV to obtain element data ranging from Mg to Ba and Ni to Bi respectively. The average resolution of the dataset is 5 cm with a measurement time of 6 seconds. Relative changes in XRF-derived elemental composition are expressed as the log ratio of two or more elements. This is common practice, as it reduces the influence of sample geometry, physical properties and matrix effect^46^. Ratios presented within this study include log(Ti/Ca) and log(Zr/Al + K). Calcium (Ca) and titanium (Ti) are conservative elements of predictable origin. Calcium represents the abundance of organic and inorganic produced marine carbonates, while titanium is an chemically inert element that is solely derived from lithogenic sources, either transported by fluvial or aeolian processes^10,12,13^. The log(Ti/Ca) may therefore be used to infer variations in terrestrial input. Aluminium (Al) and potassium (K) are primarily derived from fluvial transported aluminosilicates^10,11^. Zirconium (Zr) is enriched in heavy minerals such as zircon and rutile, which are a common constituent of windblown dust derived from the Australian desert^11,14^. The log(Zr/Al + K) can therefore be utilized to distinguish between aeolian and fluvial input of terrestrial material. Additionally, the log ratio of strontium (Sr) to calcium (Ca) has been calibrated to the XRD-derived aragonite content to create a high-resolution record of mineral changes (R^2^ = 0.77, p < 0.01, Supplementary Fig. S2). This relationship is based on the elevated strontium content of aragonite as compared to calcite^47^. Changes observed for XRF ratios or siliciclastic content are not thought to be connected to variations in sedimentation rate, since there is no clear correlation between both datasets. At 10.1 ka BP, for example, log(Ti/Ca) greatly increases while the sedimentation rate remains mostly constant (Supplementary Fig. S1).

### Radiocarbon dating

Radiocarbon age dating of planktic foraminifera, ooids and aragonite needles (n = 4) has been conducted at the Centre for Accelerator Mass Spectrometry (University of Cologne, Germany). Samples underwent treatment prior to measurement as outlined in Rethemeyer *et al*.^48^. Aragonite mud samples have been additionally wet-sieved for their fine fraction (<64 µm) to guarantee that acquired ages are representative to aragonite needle precipitation timing. SEM-imaging revealed that this is a good approximation, with the mud fraction being almost exclusively (>99%) composed of aragonite needles (Fig. 3).

Calibration of calendar years was achieved by a Bayesian age modelling approach^49^ using the Marine13 database^50^. Utilizing the marine reservoir database (http://calib.org/marine/) we averaged 10 known reservoir ages in a 1000 km radius^51,52^. The resulting reservoir age is minor (ΔR 57 ± 27) and therefore set to a local correction of 0.

The age data generated for this study complements an already existing dataset, which includes the upper 14 meters at Site U1461^17^ (Supplementary Table S1). A continuous age model was achieved by utilizing Bchron. Bchron is an age-depth modelling package implemented in “R”, using the algorithm of Haslett and Parnell^53^ (Supplementary Fig. S3). We additionally conducted an outlier analysis to assess the quality of individual data points. Resulting outlier percentages are in the sub 2% range, with most data points being below 1% (Table S1).

Ages of aragonite needles are treated to be equal to their age of formation. This assumption is based on their formation process which is interpreted to be the product of inorganic precipitation (i.e. whitings)^5^. Following precipitation, needles might be re-suspended over a matter of decades during which they experience repeated overgrowth^54^. This estimation is consistent with radiocarbon measurements which yielded average ages of present day whiting mud between 20 to 30 years^27,55^. Considering these factors, we do not expect a large age offset between dated aragonite needles and other sedimentary components (f. ex. planktic foraminifera). However, if an offset exists it would incentivize the use of aragonite mud instead of the more classical planktic foraminifera, since the later would not yield the “real” age of aragonite precipitation.

The age data is complemented by a single ooid age. The dated ooid may have formed over an extended period of time around a peloidal nucleus. Ages determined on a bulk ooid should therefore be treated carefully and has to be interpreted within the context of a larger dataset. Age measurements on stranded ooids found at the NWS yielded formation times between 1 to 1.5 kyr’s^1^. Due to the listed uncertainties the ooid age data was excluded from the creation of the continuous age model. However, the age of the measured ooid is in agreement with the general age model, strengthening the credibility of the over- and underlying data points (Supplementary Fig. S3).

## Supplementary information

Supplementary Information

Supplementary Dataset 1

## Data Availability

The data used in this article can be accessed at https://doi.pangaea.de/10.1594/PANGAEA.908697. IODP Shipboard data (Lightness and Smear Slide data) can be accessed through the LIMS webpage: http://web.iodp.tamu.edu/LORE/.
