# Supplementary material for: Increased fluvial runoff terminated inorganic aragonite precipitation on the Northwest Shelf of Australia during the early Holocene
Source: Sci Rep. 2019 Dec 4;9:18356. doi: 10.1038/s41598-019-54981-7 (PMC6892919; doi:10.1038/s41598-019-54981-7)
Supplement: Supplementary file 1 — Supplementary Information [file 41598_2019_54981_MOESM1_ESM.pdf]

SUPPORTING INFORMATION FOR

Increased fluvial runoff terminated inorganic aragonite precipitation on the Northwest Shelf of Australia during the early Holocene

Maximilian Hallenberger[1]*, Lars Reuning[2], Stephen J. Gallagher[3], Stefan Back[1], Takeshige Ishiwa[4], Beth A. Christensen[5], and Kara Bogus[6]

[1]Energy and Mineral Resources Group (EMR), Geological Institute, RWTH Aachen University, Aachen, Germany
[2]Institute of Geosciences, CAU Kiel, Kiel, Germany
[3]School of Earth Sciences, University of Melbourne, Melbourne, Australia
[4]National Institute of Polar Research, Tokyo, Japan
[5] School of Earth and Environment, Rowan University, Glassboro, New Jersey, United States
[6] Camborne School of Mines, University of Exeter, Exeter, United Kingdom

*Correspondence and requests for materials should be addressed to M.H. (email: maximilian.hallenberger@emr.rwth-aachen.de)

Supplementary Table S1. The combined radiocarbon age data of the upper ~14 m at Site U1461. Material (Mat.) used for age dating includes planktic foraminifers (F), macrofossils (M), ooids (Oo) and aragonite needle mud (AM).

Sample Name	Reported in:	Depth (m) CSF-A/CCSF	Radiocarbon age (yr BP)	95% Density Region		Mat.	Outlier Probability (%)
356-U1461C-1H-1W-9/11	Ishiwa et al. (2019)	0.1 / 0.1	1461±37	925	1084	F	1.5
356-U1461C-1H-1W-109/111	Ishiwa et al. (2019)	1.1 / 1.1	2580±46	2142	2336	F	1.4
356-U1461A-2F-2W-90/94	Ishiwa et al. (2019)	4.02 / 4.02	4850±31	5049	5257	F	0.7
356-U1461C-1H-4W-49/51	Ishiwa et al. (2019)	5 / 5	5287±33	5584	5703	F	1.6
356-U1461C-1H-6W-29/32	Ishiwa et al. (2019)	7.8 / 7.8	6015±39	6334	6517	M	1.2
356-U1461A-3F-1W-80/84	Ishiwa et al. (2019)	7.1 / 7.1	6825±35	7284	7409	F	-
356-U1461B-2H-3W-69/71	Ishiwa et al. (2019)	8.1 / 8.8	6953±36	7406	7516	F	0.9
356-U1461B-2H-3W-109/111	Ishiwa et al. (2019)	8.5 / 9.2	7574±37	7955	8119	F	1.0

356-U1461C-2H-1W-109/111	Ishiwa et al. (2019)	9.5 / 10.5	8017±41	8390	8548	F	1.1
356-U1461C-2H-2W-93/95	This Study	10.8 / 11.8	8760 +/- 40	9334	9513	F	1.9
356-U1461C-2H-3W-43/45	This Study	11.8 / 12.8	9707±84	10775	11248	AM	0.6
356-U1461C-2H-3W-93/95	This Study	12.3 / 13.3	9932±86	11204	11746	AM	1.6
356-U1461C-2H-4A-10/11	Ishiwa et al. (2019)	13.0 / 14.0	11991±55	13335	13526	M	1.4
356-U1461C-2H-4W-43/45	This Study	13.3 / 14.3	16010±50	18734	18967	Oo	-
356-U1461C-2H-4W-46/52	Ishiwa et al. (2019)	13.4 / 14.4	20157±60	23645	23923	M	0.8
356-U1461B-3H-1A-62/63	Ishiwa et al. (2019)	14.5 / 15.5	49295±816	-	-	M	0.7

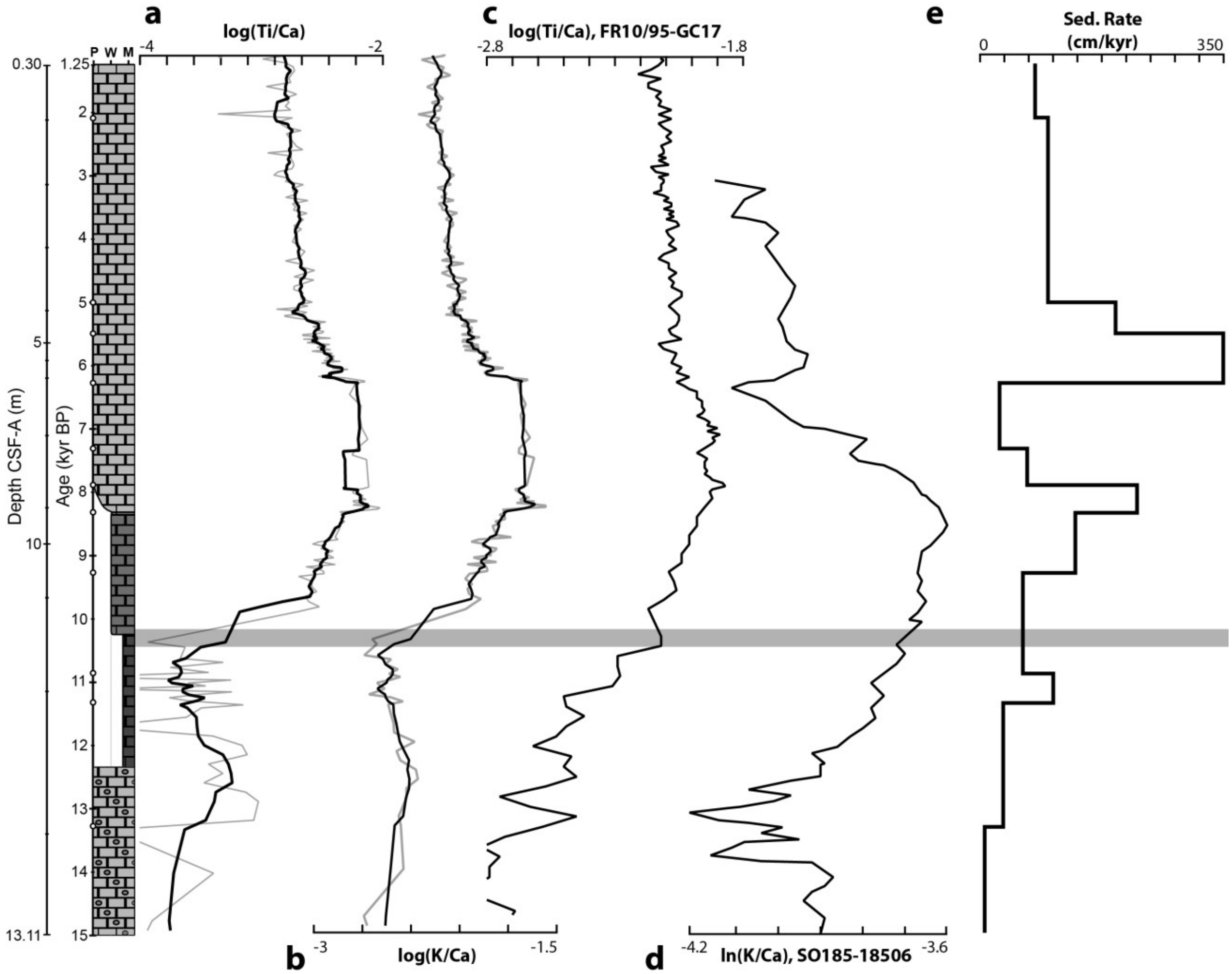

Supplementary Figure S1. Comparison of NW-Australian elemental proxy records for riverine runoff. See Figure 1 for the exact location of the sites mentioned. The grey bar underlying the

dataset marks the transition from inorganic aragonite-rich sediments to biogenic calcite-rich sediments. (a+b) Both the log ratio of (Ti/Ca) (this study) as well as the (K/Ca) ratio[15] are utilized at Site U1461 to determine the onset of humid conditions at the NWS. (c) FR10/95-GC17 riverine runoff proxy, North West Cape[10]. (d) Timor Sea (SO185-18506) precipitation record[11] (e) Sedimentation rates at Site U1461 are calculated with age data presented in Supplementary Table S1.

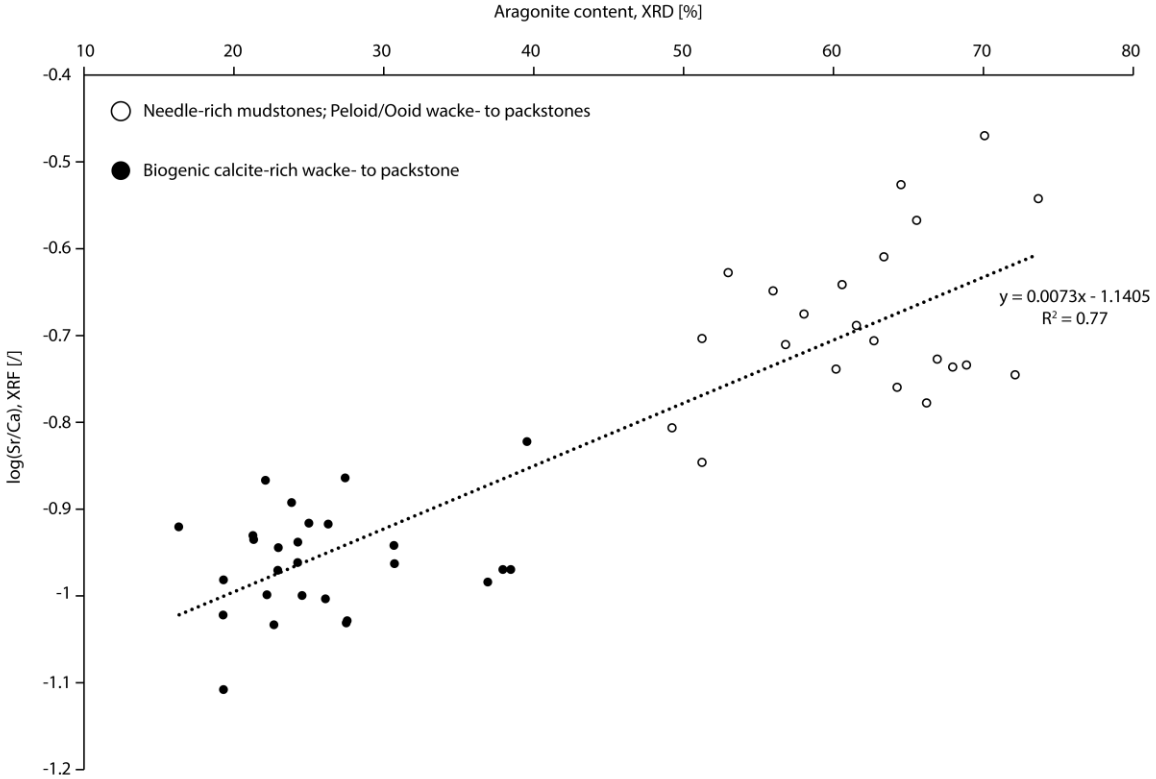

Supplementary Figure S2. Cross-plot between aragonite content (XRD) and XRF derived log ratios of (Sr/Ca) reveals a strong (R^2=0.77, p<0.01) linear relationship. This relationship is based on the elevated incorporation of strontium (Sr) within aragonite as compared to calcite. Data plots within two distinct cluster which are defined by high and low aragonite contents representing inorganic aragonite-rich sediments and biogenic calcite-rich sediments respectively. The lack of intermediate values demonstrates that inorganic aragonite production is either active or entirely absent at Site U1461.

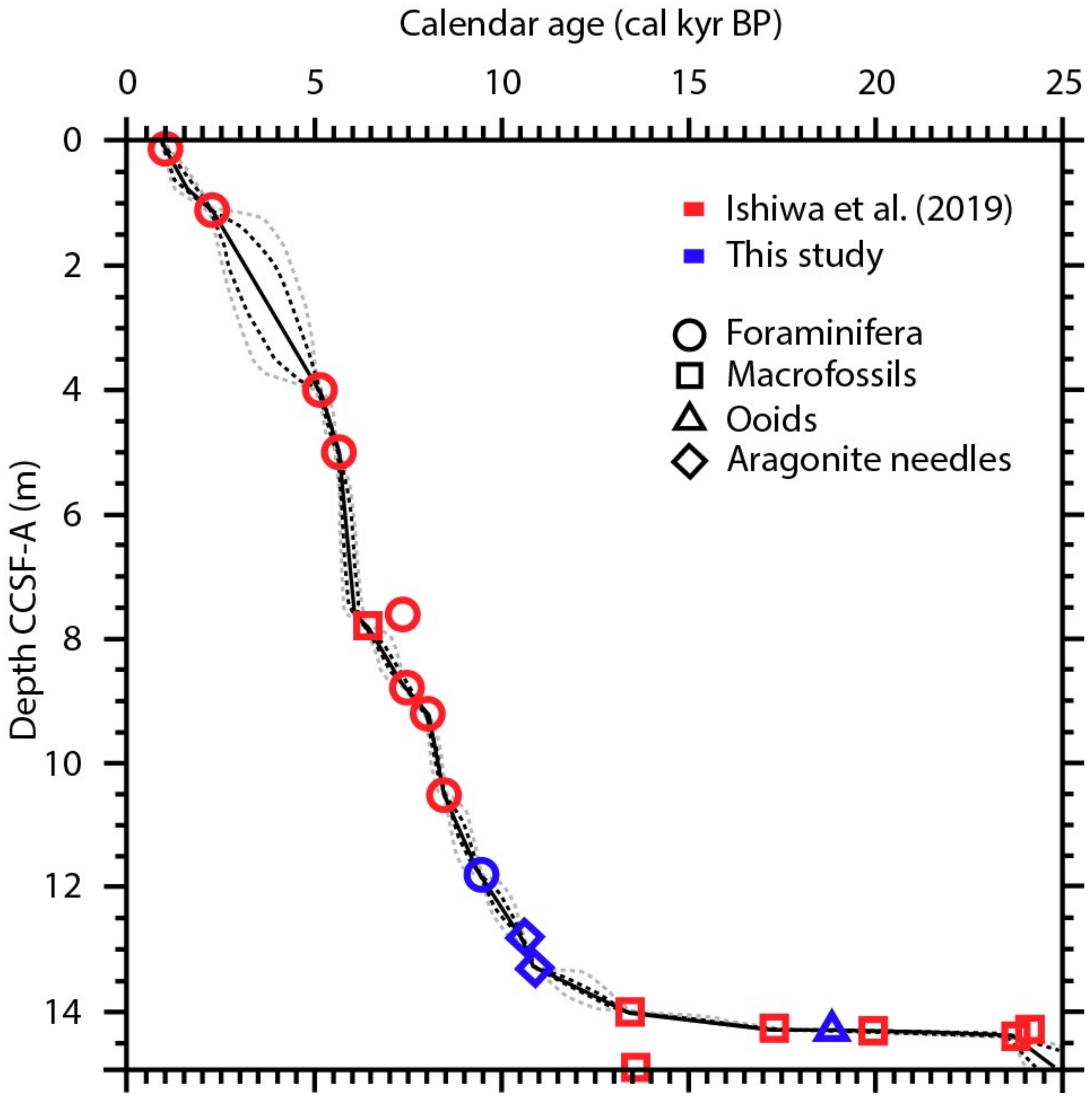

Supplementary Figure S3. Age-depth model of the upper 14 meters at Site U1461. A continuous age-depth relationship was established using the Bchron algorithm[55]. Dashed black and grey lines indicate the 90% and 97.5% confidence intervals respectively.
